# Traffic flow forecasting using natural selection based hybrid Bald Eagle Search—Grey Wolf optimization algorithm

**DOI:** 10.1371/journal.pone.0275104

**Published:** 2022-09-26

**Authors:** Sivakumar R., Angayarkanni S. A., Ramana Rao Y. V., Ali Safaa Sadiq

**Affiliations:** 1 School of Electronics Engineering, Vellore Institute of Technology, Vellore, India; 2 Department of Networking and Communication, School of Computing, SRM Institute of Science and Technology, Chengalpattu, India; 3 Department of Electronics and Communication, College of Engineering Guindy, Anna University, Chennai, India; 4 Department of Computer Science, Nottingham Trent University, Clifton Lane, NG11 8NS, Nottingham, UK; Torrens University Australia, AUSTRALIA

## Abstract

In a fast-moving world, transportation consumes most of the time and resources. Traffic prediction has become a thrust application for machine learning algorithms to overcome the hurdles faced by congestion. Its accuracy determines the selection and existence of machine learning algorithms. The accuracy of such an algorithm is improved better by the proper tuning of the parameters. Support Vector Regression (SVR) is a well-known prediction mechanism. This paper exploits the Hybrid Grey Wolf Optimization–Bald Eagle Search (GWO-BES) algorithm for tuning SVR parameters, wherein the GWO selection methods are of natural selection. SVR-GWO-BES with natural selection has error performance increases by 48% in Mean Absolute Percentage Error and Root Mean Square Error, with the help of Caltrans Performance Measurement System (PeMS) open-source data and Chennai city traffic data for traffic forecasting. It is also shown that the increasing population of search agents increases the performance.

## 1. Introduction

Traffic congestion may arise due to a lack of planned infrastructure, poor lane formation, abnormal events, scheduled overloading of vehicles during weekdays, weekend points of interest, and climate change [1]. Traffic congestion affects day-to-day life from transportation to logistics which may even lead to drip in the growth of an individual and society. However, in the era of transforming vehicles to autonomous vehicles, the accurate prediction of road traffic flow is still challenging.

Optimization algorithms are vital in estimating the ’the best’ solution from a set of solution spaces. In Optimization, a selection mechanism is then used to select individuals to be used as parents to those of the next generation. These individuals will then be crossed and mutated to form new offspring. The next generation is finally formed by an alternative mechanism between parents and their offspring [2]. This process is repeated until a specific satisfaction condition is met. Selection is the critical process in evolutionary algorithms to select healthy individuals to survive as parents for a consequent generation. Finally, the selected parents will be crossed over and mutated to form new generation individuals. Nature-inspired computing is a crucial discipline that brings to the development of novel optimization algorithms inspired by the natural behavior of flora and fauna. The most successful nature-inspired algorithms are Ant Colony Optimization [3], Particle Swarm Optimization [4], Bee algorithm [5], Grey Wolf Optimizer [6], Cuckoo search algorithm [7], and Bald Eagle Search algorithm [8].

This work builds upon the authors’ previous results on the optimal tuning of Support Vector Machine parameters by combining the hunting strategy of Grey Wolf Optimization with the fish swooping of the Bald Eagle Search Algorithm [9]. In general, the performance of any algorithm is determined by its accuracy. Therefore, the Support Vector Regression (SVR) methodology is optimized by Grey Wolf Optimization (GWO) [6] to give a more precise prediction, which, in turn, is tuned by the novel Bald Eagle Search (BES) algorithm [8]. The combination of tracking and surrounding the prey is followed by swooping the prey, which yields a more accurate prediction with faster convergence.

For any optimization algorithms, the selection is vital in attaining the best solution. In general, survival of the fittest is the basic principle. Al-Betar proposed some natural selection methods for GWO [10], and they are shown to perform better than the greedy GWO—the original GWO proposed by Mirjalili.

The remainder of this paper is organized as follows: Literature survey related to this work is given in Section 2. Section 3 narrates the hybrid GWO-BES algorithm and natural selection methods of GWO. Experimental data and graphical results are illustrated in Section 4. The conclusion and planned future research activity are given in Section 5.

## 2. Literature survey

Traffic flow is a measure of the average number of vehicles flowing in a road segment per unit time. Traffic flow forecasting and prediction were initiated with the application of Kalman filtering [11] in decades of the 80s. During the next decade, the application of seasonal time series models evolved in different parts of the world like Virginia [12], Jordan [13], Texas [14], and Germany [15]. Neural networks were used to predict short-term traffic flow casting [16]. Artificial Intelligent and Machine learning algorithms are continuously working on traffic prediction, traffic flow forecasting, traffic sign detection, and traffic accident analysis. Deep Neural Network Based Traffic Flow model has been proposed by Wu et al. [17] for the prediction of traffic flow with the attention-based model. Time Series based analysis found wide application for traffic forecasting [18, 19]. Statistics-based prediction methods are working linear regression models [20], multivariate nonparametric regression [21], and K-Nearest Neighbors [22]. The autoregressive model was introduced to predict short-term traffic flow with the limited data as input [23]. A detailed study on different methodologies for short-term forecast of road traffic data has been studied in [24]. Various categories of traffic congestion detection schemes and tools have been illustrated [25].

Support Vector Regression (SVR) has been used for a long time in the Intelligent Transportation domain in combination with various algorithms. Wu et al. [26] applied the SVR for travel time prediction with the thirty-five days’ data of vehicle speeds collected from loop detectors in Taiwan city. An incremental SVR model has been proposed [27], which proves better than Back Propagation Neural Networks. SVR finds its application in the prediction of bus travel time [28]. The Tabu search method was combined with the SVR for forecasting highway traffic [29]. SVR has been used in combination with other evolutionary algorithms to improve accuracy.

Grey wolves (Canis lupus) usually live as a pack in the wildlife. Inspired by the Grey wolves’ behavior in social relationships and leadership, the Grey Wolf optimization technique was proposed by Mirjalili [6]. Grey Wolf Optimization (GWO) has found a wide range of applications like resolving power dispatch issues [30], risk prevention in smart grid [31], solving economic issues [32] and price bidding [33], Parkinson’s disease identification [34] and IoT Botnet detection [35]. From then, it became one of the most successful and best nature-inspired computing. Many variations of GWO are evolving in recent years, including modifications of the operators, hybrid combination with another optimization technique [36], improved exploration of than standard version [37], fuzzy logic-based dynamic parameter adaptation [38], distributed GWO [39], grouped GWO [40], adaptive randomization with GWO [41], weighted distance updating GWO [42], GWO with hierarchical operator [43] and usage of evolutionary population dynamics to improve GWO [44].

In addition to modifying the basic grey wolf optimization, there emerge many hybrid combinations of the grey wolf algorithm with other successful evolutionary algorithms. A hybrid combination of Particle Swarm Optimization with GWO (PSO-GWO) was proposed as a novel approach to optimize a single-unit commitment problem [45]. Laplace function for Support Vector Classification has been used with Grey Wolf Optimization for clustering the intruder attacks [46].

Metaheuristic algorithms such as Improved salp swarm algorithm [47], binary emperor penguin optimizer [48], Heap-based Optimizer [49], adaptive grey wolf optimizer with local search [50], Gaze Cues Learning-based Grey Wolf Optimizer [51], Migration-Based Moth-Flame Optimization Algorithm [52] have been employed to solve many engineering problems.

Differential Evolution, in combination with GWO, was applied to optimize the continuous problems and also compared with benchmark functions [53]. To solve more complex and significant optimization problems, T.S. Pan devised a parallelized strategy to divide the population of grey wolves and handle each unit of the population with GWO separately [54]. Mirjalili [55] devised a multi-objective Grey Wolf Optimization, suitable for handling multiple criteria based on real-time problems. Various natural-based selection methods [10] were proposed to enhance the performance of GWO.

## 3. Hybrid combination of BES with natural selection based GWO

### 3.1. Grey Wolf optimization for support vector regression

Support Vector Regression (SVR) is one of the successful techniques used for time series prediction problems. Due to the low complexity of SVR [56], it has a broad range of applications. For the prediction of traffic flow, traffic datasets are defined as T = {(x_i_, y_i_)}_i = 1,2,3,…N_, where N defines the number of samples taken into consideration, and x_i_ and y_i_ are defined in a multi-dimensional space (Eq (1)).


$$g(i)=W\emptyset_{i}+B$$ (1)

W and B are the weight vector and the bias value, which are mapped together with training data using the nonlinear function ∅_i_. The objective function of SVR can be mathematically given as in Eq (2).


$$F=\frac{1}{2}W^{2}+C\frac{1}{N}\sum_{k=1}^{N}L_{\varepsilon }(y_{i},g(x_{i}))$$ (2)

The original value is given by yi, whereas g(x_i_) refers to the predicted value, a constant C and ε − insensitive loss function is used to assess the error performance (Eq (3)).


$$L_{\varepsilon }=\left\{ \begin{array}{l} \;|g(i)-y|-\varepsilon \;(g(i)-y)\geq \varepsilon \\ 0\;otherwise \end{array}\right.$$ (3)

Gaussian Radial Basis Function (Eq (4)) is one of the widely used kernel models used to associate nonlinear and linear data. The kernel function is given as

$$K(x,x_{k})=exp\;(-\frac{x-x_{k}}{2\gamma^{2}})$$ (4)

The performance accuracy of SVR is defined by the parameters C, ɛ, and γ. To increase the accuracy of prediction, the Grey Wolf Optimization algorithm has been chosen to optimize the parameters of SVR.

The wild animal Grey wolf follows a unique social hierarchy and devised strategies for hunting as a group. The hierarchy of wolves from top to bottom is A, B, D, O which represents Alpha, Beta, Delta, and Omega. The most dominant one to lead the entire pack and make a decision regarding selecting habitat and prey is Alpha. The next subordinate who helps Alpha for taking decisions is Beta, which thereby rules the rest of the pack in the absence of Alpha. Third-ranking wolves are called Delta, which are multiple role players like scouts, caretakers, and sentinels and are comprised of aged members. The rest of the wolves are scapegoats of the pack, commonly called Omega.

**Fig 1:**
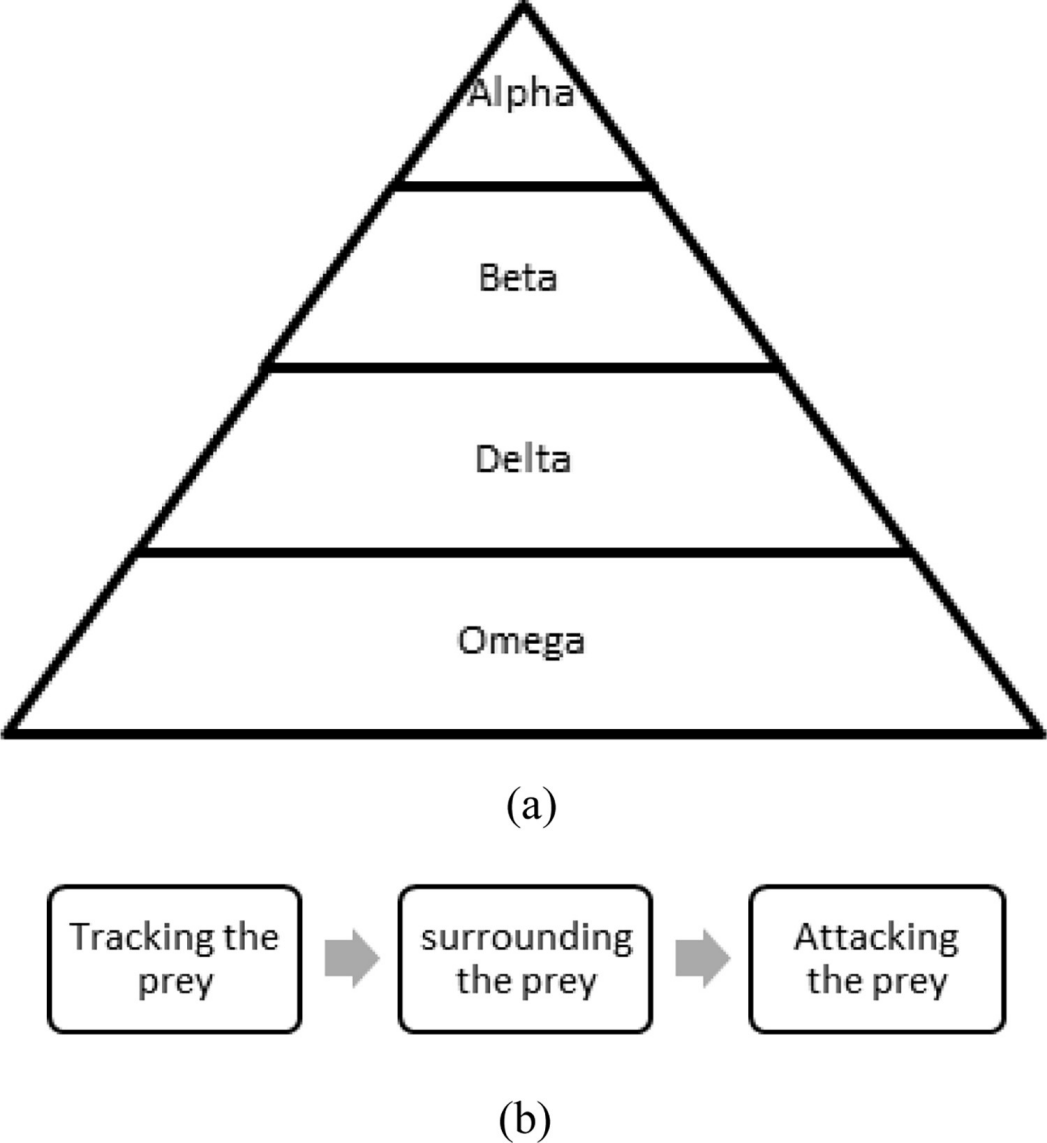
GWO (a) social hierarchy; (b) Process flow.

The hunting phases of Grey Wolves include tracking the prey, surrounding the prey, and finally attacking the prey. As an evolutionary algorithm, GWO chooses the prime fittest agents, Alpha, Beta, and Delta, in the way of hierarchy. All other remaining agents are Omega, as shown in Fig 1. Mathematically, to model the GWO, the position of the Wolf is *W*, the prey is *W*_*p*_, and the top three agents are *W*_*A*_, *W*_*B*_ and *W*_*D*_. Next following position of the wolf in a timeline is represented as given in Eq (5):

$$W(t+1)=W_{p}(t)-S.D$$ (5)

S defines the vector which changes per the direction of prey as shown in Eq (6)

$$S=K|W_{p}(t)-W(t)|$$ (6)

Coefficient vectors D and K can be expressed as

$$D=2.a.z_{1}-a$$

$$K=2.z_{2}$$

$$a=2-i\;\left(\frac{2}{M}\right)$$ (7)

where *z*_1_ and *z*_2_ take out any random values from 0 to 1.

Grey wolves finish the hunt by attacking the prey when it stops moving. While approaching the prey, initially, the wolves start moving randomly until it comes closer to the prey. Then the randomness reduces, which means all other wolves start focusing on the position of Alpha, Beta, and Delta. The randomness of the wolves’ movement is determined by the parameter ’*a*, which linearly drops from 2 to 0. The parameter ’*a*” is random but is vital in controlling the movement of wolves towards the prey. It depends on the current iteration count (i) and the maximum number of iterations (*M*), as shown in Eq (7). While searching the prey (Eq (8)), the wolves move to different positions according to the equations defined as

$$S_{A}=|K_{1}.W_{A}-W|$$

$$S_{B}=|K_{2}.W_{B}-W|$$

$$S_{D}=|K_{3}.W_{D}-W|$$ (8)

The hunting phase is expressed in Eq (9). *W*_1_, *W*_2_, and *W*_3_ are the position of a wolf when it updates its position concerning the position of Alpha, Beta, and Delta (W_A_, W_B_, and W_D_), respectively.


$$W_{1}=W_{A}-D_{1}(S_{A})$$

$$W_{2}=W_{B}-D_{2}(S_{B})$$

$$W_{3}=W_{D}-D_{3}(S_{A})$$ (9)

Grey wolf Optimizer reaches its final stage (Eq (10)) by updating the average position of the three principal wolves.


$$W(k+1)=\frac{\left(W_{1}+W_{2}+W_{3}\right)}{3}$$ (10)

### 3.2. Hybrid GWO-BES algorithm

A novel nature-based computing algorithm is the Bald Eagle Search Algorithm proposed by Alsattar [8]. Bald Eagles show the unique behavior of hunting their food, which shows their intelligence. They framed a strategy like searching for the location of prey, choosing the prey, and dive down to hunt the prey (swooping). By exploiting the speed of the wind and airflow, they are using a smart strategy of hunting.

Eagles remember the search space of prey from their previous hunting. Once the search domain is picked up, the eagle progresses towards the domain and explores to select its prey (usually salmon fish). Every movement of an eagle is governed by its previous motions and is spiral in Nature. The position of an eagle is referred to by positional difference by *r*′ from the range between 1.5 and 2, randomness is introduced by *R* in the range between 0 and 1 in (Eq (11)).


$$W_{new,k}=W_{best}+r^{\prime }*R(W_{mean}-W_{k})$$ (11)

As given in Eq (12), the best position from past hunting is represented by *W*_*best*_, and an average of previous space domains is given by *F*_*best*_. Updating of hunting the prey is given in Eqs (13) and (14).

$$W_{k,new}=W_{k}+y(k)*(W_{k}-W_{k+1}+x(k)*(W_{k}-W_{mean}))$$ (12)

$$x(k)=\frac{xs(i)}{max\;(|xs|)}$$

$$y(k)=\frac{ys(k)}{max\;(|ys|)}$$ (13)

$$xs(k)=D(k)*sin\theta_{k}$$

$$ys(k)=D(k)*cos\theta_{k}$$

$$\theta_{k}=r^{\prime \prime }*\pi$$

$$S(k)=\theta_{k}+c*RV$$ (14)

where the parameter *r*′′ is within 5–10, which determines the corner position, *c* represents the search cycle count within the values 0.5–2, *rv*, a random variable, introduces randomness, and *c*_1_ and *c*_2_ randomly picks values between 1 and 2. The final swooping stage can be formulated as in Eq (15),

$$W_{k,new}=rv*W_{best}+xt(k)*(W_{k}-c_{1}*W_{mean})+yt(k)*(W_{k}-c_{2}*W_{best})$$ (15)

$$xt(k)=\frac{xs(k)}{max\;(|xs|)}$$

$$yt(k)=\frac{yski)}{max\;(|ys|)}$$

**Fig 2:**
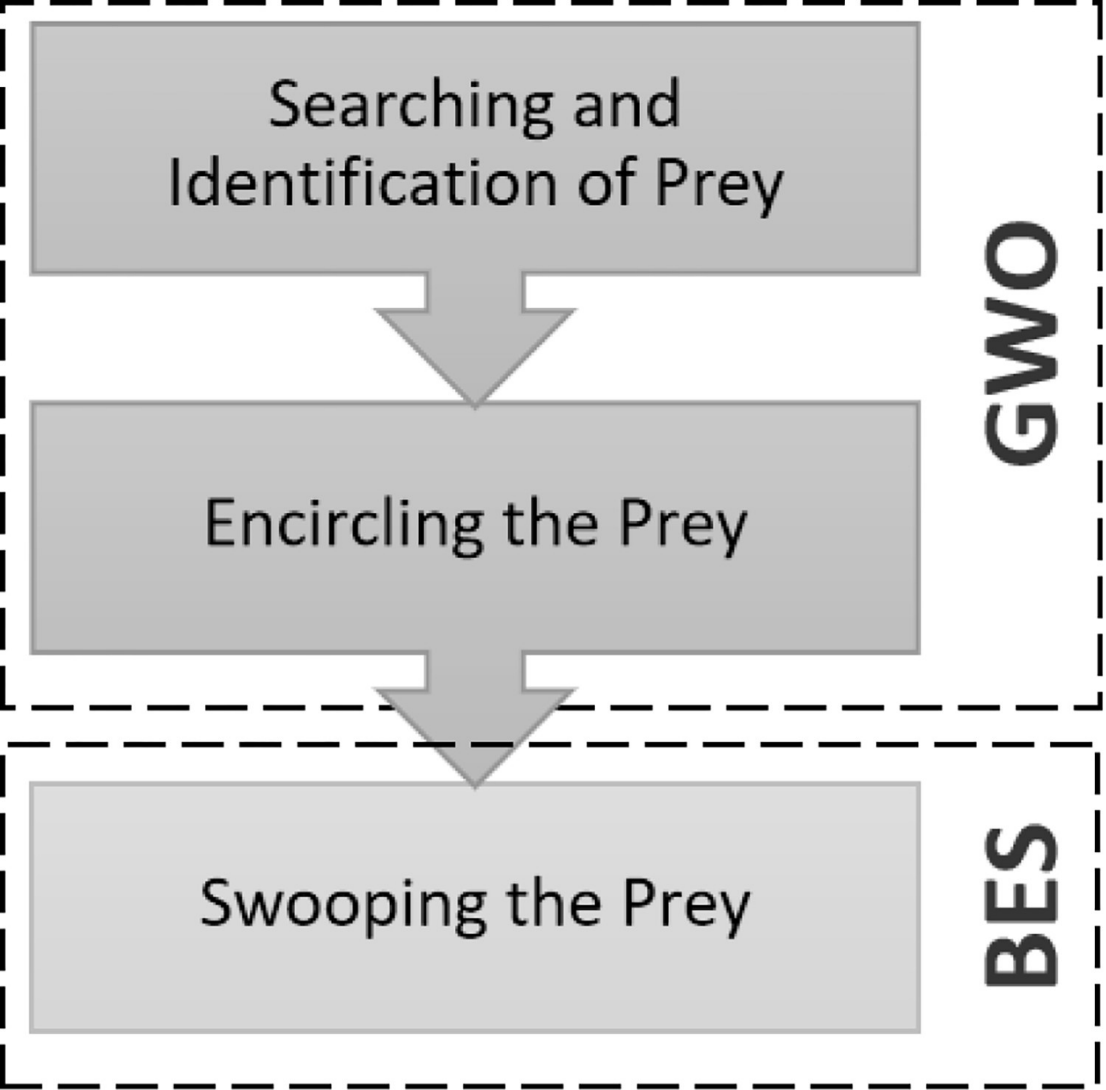
Hybrid GWO-BES.

It has been shown that a hybrid combination of exploration and exploitation of these two successful optimization algorithms: the Grey Wolf Optimizer and Bald Eagle Search algorithm, produces better prediction and faster convergence [9]. After tracking and surrounding the prey, the attacking stage of the former algorithm is replaced by the swooping phase of the latter one, as shown in Fig 2.

### 3.3. Natural selection based Hybrid SVR-GWO-BES

The Grey Wolf optimization is influenced by the three better solutions–Alpha, Beta, and Delta. Sometimes, in the process of Optimization, average solutions may lead to global optima than moving towards the best solutions. This principle results in the development of more selection methods for GWO. Azmi Al-Betar [10] proposed five different natural selection methods to use for GWO, namely–proportion-based GWO (PGWO), Tournament based GWO (TGWO), and Universal sampling-based (UGWO), Linear Rank based GWO (LGWO), Random based GWO (RGWO). The original methodology of selection defined by Mirjalili et al. [6] is named Greedy GWO. It selects the top three solutions, W_A_, W_B_, and W_C,_ which have an equal chance of surviving to reach the optimal solution. However, it fails to give a chance to other agents, which may also lead to a solution at a faster rate.

**3.3.1. Random-based GWO (RGWO).** The top-tier grey wolves in the hierarchy are randomly picked up from the current population. When contrast to other revisions of GWO, RGWO gives less accuracy and slower convergence. The probability of selection (Eq (16)) of one wolf is defined by

$$p(W_{k})=\frac{1}{K}\;\forall_{k}=(1,2,\ldots K)$$ (16)

**3.3.2. Proportional-based GWO (PGWO).** In proportional-based GWO, the selection probability of choosing one wolf is based on its absolute fitness value of all wolves. This method does not neglect any wolf from being in the selection. The probability is defined in Eq (17)

$$p(W_{k})=\frac{f(W_{k})}{K\sum_{m=1}^{K}f(W_{m})}$$ (17)

#### 3.3.3. Linear rank based GWO (LGWO)

To get the better of the proportional selection method, linear rank, which is defined by Baker [57], is used.

#### 3.3.4. Universal sampling-based (UGWO)

The Stochastic Universal Sampling method is similar to the proportional-based GWO, whereas selection is based on Baker’s proposal [58]. The probability of each wolf being survived to the next level depends on its fitness function with the combined fitness function of every wolf. The primary wolf is chosen based on selection probability. The next two wolves are divided equally from the selected first wolf.

#### 3.3.5. Tournament-based GWO (TGWO)

Over a few decades ago, Goldberg et al. [59] proposed the selection methods. These mechanisms were predominately in evolutionary algorithms such as fuzzy intelligent algorithms [60], bat-inspired algorithm [61], Modified Particle Swarm Optimization for Support Vector Machines [62], Whale Optimization algorithm in combination with simulated Annealing [63] and cuckoo search algorithm [7] and Bat algorithm [64].

**Fig 3:**
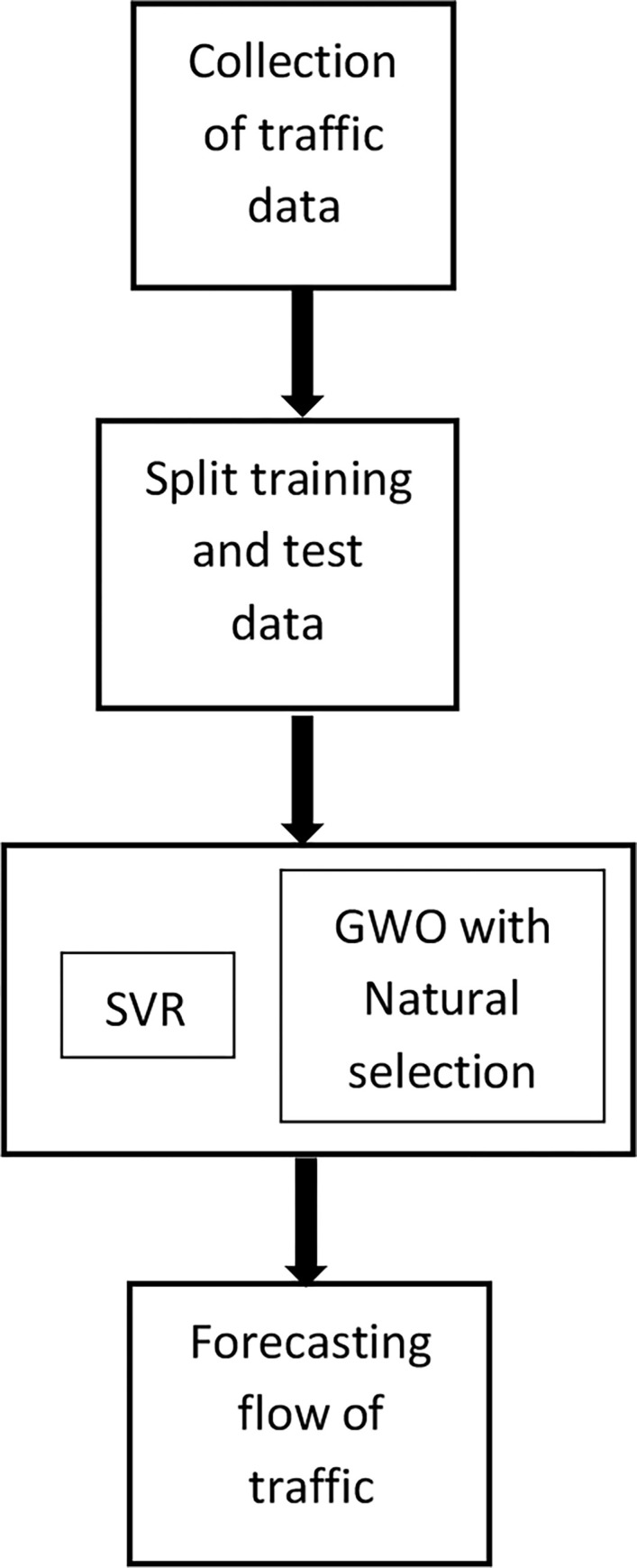
Hybrid SVR-xGWO-BES for traffic forecasting.

These natural selection methods were shown to enhance the convergence of Grey Wolf Optimizer. These methods are applied to the proposed hybrid Grey Wolf optimization–Bald Eagle Search Algorithm (Table 1). SVR–x–GWO is the term used to refer the SVR employed with natural selection based GWO (original Greedy GWO). SVR–x–GWO-BES is the term used to refer the SVR employed with hybrid GWO-BES where selection is of natural methods (Fig 3).

**Table 1:** Algorithm for the proposed work.

*Initialize the wolf population as Xi*, *where i = 1*,*2*,*…m*
*Initialize a*, *G*, *M and Max*
*Compute the fitness value of each search agent*
*X*_*A*_ *= first best search agent*
*X*_*B*_ *= second search agent*
*X*_*D*_ *= third best search agent*
*Until k reaches maximum number of iterations*
*For each search agent*
*Update the position of the search agent*
*Hunting movement*
*X*_1_ = *X*_*A*_−*G*_1_ (*D*_*A*_)
*X*_2_ = *X*_*B*_−*G*_2_ (*D*_*B*_)
*X*_3_ = *X*_*D*_−*G*_3_ (*D*_*D*_)
*Natural selection based methods to choose X*_*K+1*_
*end for*
*Update a*, *G and M*
*Calculate the fitness of all search agents*
*Update X*_*A*_, *X*_*B*_ *and X*_*D*_
*k* = *k*+1
*end while*
*return X*_*A*_

## 4. Results and discussion

The datasets used for the analysis are lane-disciplined road data from California and lane-less road traffic data from India. The department of transportation of California is organizing and distributing road traffic volumes with a web interface [65]. The selected datasets are similar to that used in the proposal of a hybrid GWO-BES algorithm [9]. Milgeo Avenue of Northbound of California is used as source data. In March 2016, data was used as training data and testing data, respectively. This is an example of lane-based traffic volumes in a part of California. For lane-less traffic volumes, the major junction at Chennai city road traffic [66] is chosen, an example of Asian roads. Few major intersections in Chennai city are monitored by the Traffic Regulation Observed Zone (TROZ), a joint venture of Greater Chennai Traffic Police, Hyundai Motors India Foundation, Alco Systems, and Videonetics. The primary motive of TROZ is the automatic detection of traffic rule violations, but they also collected the traffic flow at the monitored intersections at regular intervals.

For analysis purposes, Random based GWO (RGWO), Universal sampling-based GWO (UGWO), Proportional based GWO (PGWO), Linear rank based GWO (LGWO), and Tournament based GWO (TGWO) are applied with PeMS 2016 dataset and TROZ dataset. Likewise, earlier proposed GWO-BES is applied to the above five natural selection-based GWO.

**Fig 4:**
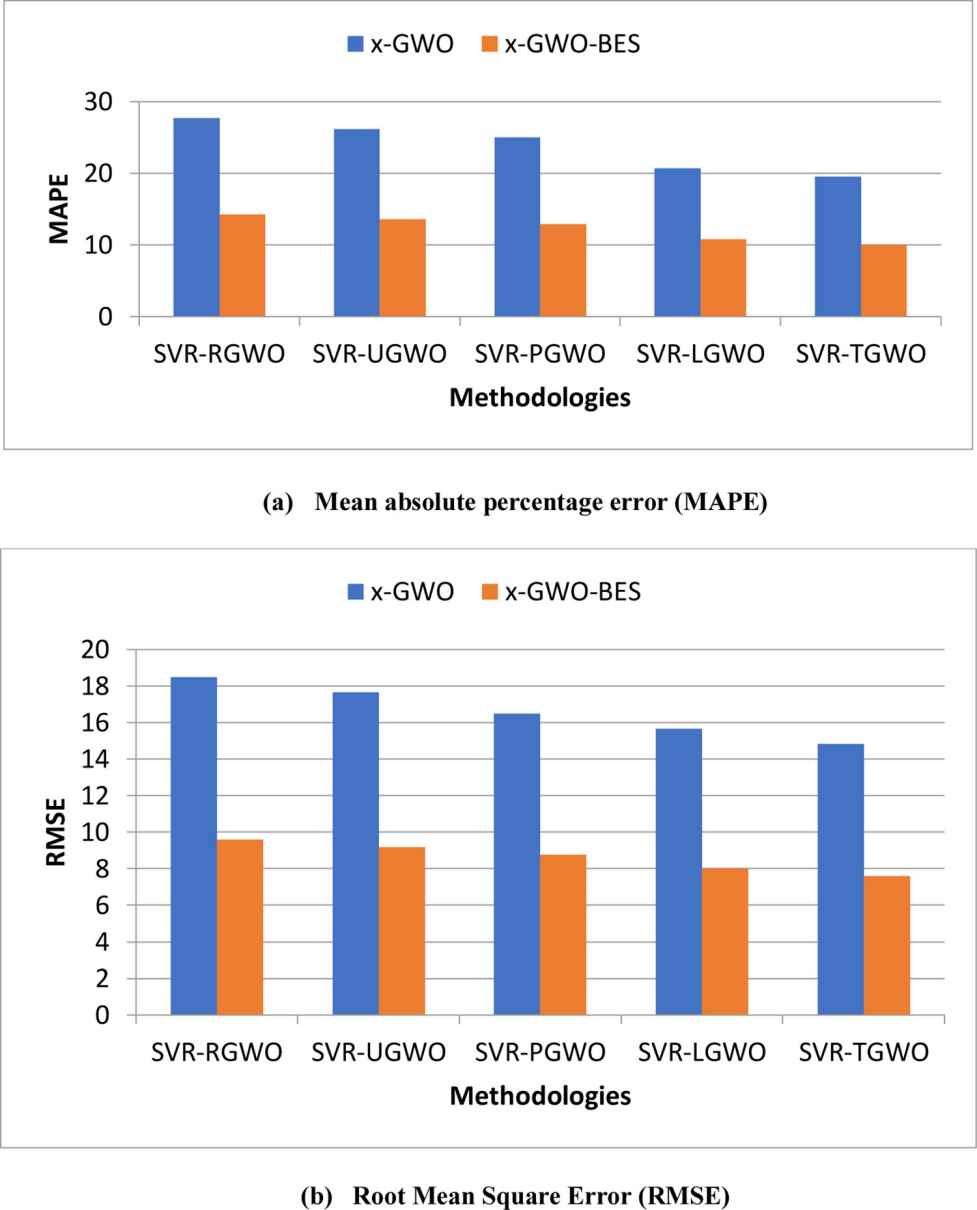
Analysis of hybrid GWO-BES on natural selection based GWOs (a) MAPE (b) RMSE.

For the chosen dataset, the error performance for the prediction has been shown in Fig 4. It clearly indicates that the hybrid combination of the Bald Eagle Search Algorithm with Grey Wolf Optimization results in faster convergence. It helps in providing more accurate predictions, thereby reducing the error performance (Table 2). This gives clear indication that hybrid GWO-BES can be used to various evolved versions of GWO with reasonable accuracy. This may increase the feasibility of applying this scheme for real-time data prediction. The parameters of SVR, like C, € and γ, which determines the accuracy of the regression, takes the optimized values as 0.2123, 0.0247 and 0.0236 respectively.

**Table 2:** Error measure for traffic prediction for proposed SVR–x–GWO-BES and SVR–x–GWO—MAPE and RMSE.

MAPE	x-GWO	x-GWO-BES
**SVR-RGWO**	27.67	14.25
**SVR-UGWO**	26.17	13.58
**SVR-PGWO**	25.00	12.92
**SVR-LGWO**	20.67	10.75
**SVR-TGWO**	19.50	10.00
**RMSE**	**x-GWO**	**x-GWO-BES**
**SVR-RGWO**	18.50	9.58
**SVR-UGWO**	17.67	9.17
**SVR-PGWO**	16.50	8.75
**SVR-LGWO**	15.67	8.00
**SVR-TGWO**	14.83	7.58

### 4.1. Impact of search agents

The population of search agents used for Optimization is varied, and its impact on prediction is studied in this paper. Initially, the number of search agent wolves chosen is 30. The maximal number of iterations is limited to 5. The range of some search agents chosen is 10, 15, 20, 25, and 30. When the number of search agents is increased, it gives more accuracy in prediction; that is, the error rate decreases. The mean absolute percentage error (MAPE) is reduced from 23.17 to 18.50 when the number of search agents is increased from 10 to 30, as shown in Table 3(A). Likewise, RMSE dropped from 11.92 to 9.58, as the number of search agents increased from 10 to 30, as shown in Table 3(B).

**Table 3:** Error measure for traffic prediction for increasing search agents–(a) MAPE and (b) RMSE.

	Number of Search agents				
Algorithm	10	15	20	25	30
**(a) MAPE**					
**SVR-RGWO_BES**	23.17	22.00	21.50	20.67	18.50
**SVR-UGWO_BES**	22.83	21.83	21.17	20.83	17.67
**SVR-PGWO_BES**	22.50	21.33	20.50	19.67	16.50
**SVR-LGWO_BES**	22.00	21.00	20.00	19.17	15.67
**SVR-TGWO_BES**	21.50	20.67	19.83	19.00	14.83
**(b) RMSE**					
**SVR-RGWO_BES**	11.92	11.33	11.08	10.67	9.58
**SVR-UGWO_BES**	11.75	11.25	10.92	10.75	9.17
**SVR-PGWO_BES**	11.75	11.17	10.75	10.33	8.75
**SVR-LGWO_BES**	11.17	10.67	10.17	10.01	8.00
**SVR-TGWO_BES**	10.92	10.50	10.08	9.67	7.58

## 5. Conclusion

Traffic flow forecasting, which is one of the critical challenges in transportation, has been taken into account with the aid of the most popular prediction algorithm–support vector regression. Hybrid GWO-BES proved to produce better accurate predictions for the greedy GWO. This paper shows that the hybrid GWO-BES algorithm can also be used to optimize the natural selection method-based GWO like Random GWO, Universal Sampling based GWO, Proportional based GWO, Linear rank based GWO, and Tournament based GWO. SVR-xGWO-BES reduces MAPE and RMSE by approximately 48%. Experimental results show that the size of the population of grey wolves has an impact on prediction accuracy. An increasing number of search agents produce reduced error performance. In a pack of grey wolves, although the alpha pair is only allowed breeding pair, the female alpha wolf is not only involved in breeding but also supports the male alpha wolf in the hunting phase. For future work, it is intended to work on the GWO algorithm by introducing Alpha pair rather than single Alpha in hunting. This hybrid GWO-BES can also be used to analyze the variations of traffic flow in less-lane disciplined road traffic data with heterogeneous vehicles.
